# Neuralgia of the Heart

**Published:** 1831

**Authors:** 


### XLII.

### NEURALGIA OF THE HEART.

DR. ELLIOTSON mentions the case of a man who had been discharged from St. Thomas's Hospital, little, if at all relieved, and who appears to have laboured under an affection of the kind in question. He had sudden pain recurring across the chest, diagonally from the centre of the sternum across the left nipple. It was not more violent when walking gently than when at rest—but if he moved quickly the pain was more severe. Nothing particular was observable in the pulse, nor in the chest, when examined by the stethoscope. The pain was sudden and transient—darting like neuralgia in other parts. "It was not (says Dr. E.) angina pectoris, for it came on as frequently when he was sitting perfectly still, and he even got relief from gentle motion. It did not stop his breath at all; nor make him feel faint, or give him a dying sensation." Dr. E. gave him the carbonate of iron, and he was much better in some respects—that is to say, he was free from pain so long as he kept quiet—but it recurred when he moved about. He would not remain in the hospital for sufficient trial. Taking the foregoing circumstances into consideration, we are inclined to differ (which we seldom do) from our friend Dr. E. We think it was one of the many forms which angina pectoris assumes. It has been designated by the term steno-cardia—sternalgia, and many other names; nor is it essential that the paroxysm should be brought on by muscular exertion. We have seen many instances where this was not the case, yet where the disease was evidently angina pectoris. Even in the above case, the exertion of the muscles, when arrived to a certain amount, excited the paroxysm. We are inclined to coincide with Dr. E. that the complaint is a species of neuralgia.

---

